# 

**Published:** 1911-03-04

**Authors:** 


### CONTENTS.

| | PAGE |
|---|---|
| **Doctors and International Peace** | 647 |
| **The General Practitioner—His Book** | 648 |
| **Annotations—** The First Operation under Ether—The Treatment of Yaws with "606"—A New Source of Rubber | 649 |
| **Medical Opinion and Movement** | 650 |
| **Hospital Clinics—** Strangulated Hernia. By Owen Richards, M.Ch.Ox., F.R.C.S. | 653 |
| **Practical Notes on Diagnosis and Treatment** | 655 |
| **Special Articles—** The Bubonic Plague and Chinese Remedies | 656 |
| **Neurology—** 1.—Some Spinal Cord Lesions | 657 |
| **Anæsthetics—** Sweating and Anæsthesia | 658 |
| **News and Events of the Week** | 659 |
| **Obituary** | 660 |
| **Medical Appointments, &c.** | 660 |
| **The Week in the Courts** | 660 |

| | PAGE |
|---|---|
| **The Week's Work—** The New King's College Hospital—The Future—A Popular Exposition of Hospital Work—The Institutional Quarterly... | 661 |
| For Hospital Readers—The Week's Drug Market | 662 |
| **The Hospital World** | 662 |
| **Special Institutional Article—** A Controlling Authority for Voluntary Hospitals | 663 |
| **Royal Portsmouth Hospital** | 665 |
| **Jottings** | 666 |
| **Gifts and Bequests** | 666 |
| **Institutional Notes and News** | 667 |
| **The New King's College Hospital, Denmark Hill** | 668 |
| **The Late King and the Hospitals** | 669 |
| **Institutional Letter Box** | 670 |
| **Parliament and Publications** | 671 |
| **New Appliances and Things Medical** | 672 |
| **The Week's Novelties** | 672 |
| **Answers to Correspondents** | 672 |
| **Buildings Contemplated and in Progress** | 672 |
| **Medical Appointments** | 672 |

